# Collagen peptides supplementation improves function, pain, and physical and mental outcomes in active adults

**DOI:** 10.1080/15502783.2023.2243252

**Published:** 2023-08-08

**Authors:** Shiloah A. Kviatkovsky, Robert C. Hickner, Hannah E. Cabre, Stephanie D. Small, Michael J. Ormsbee

**Affiliations:** a Florida State University, Department of Nutrition and Integrative Physiology, Tallahassee, FL, USA; b Florida State University, Institute of Sports Sciences and Medicine, Tallahassee, FL, USA; c University of Arkansas for Medical Sciences, Center for Aging and Longevity, Geriatrics, Little Rock, AR, USA; d The University of North Carolina at Chapel Hill, Applied Physiology Lab, Department of Sport Science, Chapel Hill, NC, USA; e University of Toronto, Faculty of Kinesiology & Physical Education, Toronto, ON, Canada; f University of KwaZulu-Natal, School of Health Sciences, Discipline of Biokinetics, Exercise and Leisure Sciences, Durban, South Africa

**Keywords:** Gelatin, hydrolyzed, KOOS, VR-12, nutrition, connective tissue

## Abstract

**Introduction:**

Chronic pain affects 19% of adults in the United States, with increasing prevalence in active and aging populations. Pain can limit physical activity and activities of daily living (ADLs), resulting in declined mental and social health. Nutritional interventions for pain currently target inflammation or joint health, but few influence both. Collagen, the most abundant protein in the human body and constituent of the extra cellular matrix, is such a nutraceutical. While there have been reports of reductions in pain with short-term collagen peptide (CP) supplementation, there are no long-term studies specifically in healthy middle-aged active adults.

**Purpose:**

To determine the effects of daily CP consumption over 3, 6, and 9 months on survey measures of pain, function, and physical and mental health using The Knee Injury & Osteoarthritis Outcomes Score (KOOS) and Veterans Rand 12 (VR-12) in middle-aged active adults.

**Methods:**

This study was a double-blind randomized control trial with three treatment groups (Placebo, 10 g/d CP, and 20 g/d CP).

**Results:**

Improvements in ADLs (*p* = .031, η_p_^2^ = .096) and pain (*p* = .037, η_p_^2^ = .164) were observed with 10 g/d CP over 6 months, although pain only improved in high frequency exercisers (>180 min/week). Additionally, VR-12 mental component scores (MCS) improved with 10 g/d of CP over 3–9 months (*p* = .017, η_p_^2^ = .309), while physical component scores (PCS) improved with 20 g/d of CP over 3-9 months, but only in females (*p* = .013, η_p_^2^= .582).

**Conclusion:**

These findings suggest 10 to 20 g/d of CP supplementation over 6 to 9 months may improve ADLs, pain, MCS, and PCS in middle-aged active adults.

## 1. Introduction

Aging is accompanied by declines of physiological processes related to tissue regeneration in skin, muscle, connective tissue, and bone. Degeneration of these tissues commonly result in decreased physical function, injury, and subsequent long-term pain and disability [1,2]. Approximately 19% of U.S. adults suffer from chronic pain, often caused by musculoskeletal injury, of which 50% involve tendons or ligaments [3,4]. This amounts to an estimated $600 billion burden on the healthcare system annually [3,4]. Therefore, it is imperative to evaluate low cost and time commitment interventions for preventing injury and managing chronic pain.

Collagen is the most abundant protein in the human body and a predominant component of tendons and ligaments, that unfortunately degrades disproportionately with age compared to muscle tissue and bone. Onset of age-related losses of collagen begins as early as the second to third decade of life, with reductions of approximately 1% per year after 40 years of age [1,4,5]. This trajectory can result in total body collagen losses of up to 75% by 80 years of age [1,4,5]. In addition, the loss of collagen, especially in tissues exposed to the greatest forces (e.g. tendon, ligaments), may result in connective tissue impairments and injury [2,6].

Although physical activity is largely recommended to improve health and prevent age-related declines in body tissue, prevalence of chronic pain increases with age and activity level, especially in females compared to males [7]. Those who experience pain tend to exhibit pain avoidant behaviors, which result in decreased physical activity that can also prompt pain. This creates a cycle of physical activity restriction that can not only lead to increased disability and decreased participation in sport, but also impairments in activities of daily living (ADLs) [5,8]. Furthermore, pain as well as pain avoidant behaviors can diminish quality of life (QOL) and deteriorate mental, social, and economic determinants of health, especially in aging active populations [5,8].

Pharmacological interventions for chronic pain management consist of drugs that reduce pain and inflammation, such as NSAIDs and corticosteroids. While effective short term, this modality of pain management can have drastic side effects when taken long term, such as gastrointestinal tract, nerve, and connective tissue injury [9–12]. Additionally, these pharmacological treatments do not target or correct the etiology of the pain. Therefore, alternative treatments without side effects that target both the etiology and symptom of pain, are needed.

Collagen peptides (CP) are a well-established supplement in the skin and anti-aging industry for their beneficial effects on skin elasticity and hydration as well as reversing photodamage from UV radiation [13]. More recently, a growing body of research has emerged in support of improvements in joint pain, inflammation, ADLs, and return to play after injury with CP supplementation [14–19]. These benefits could be a result of increased substrate for building new collagen fibers, or through signaling cascades resulting in increased connective tissue turnover. However, these mechanisms have not yet been elucidated. While data from these studies support the use of CP supplements to reduce pain and time back to sport, this research has only been done in young athletic populations, those with specific joint injuries or osteoarthritis, and those participating in rehabilitative programs for injuries. Consequently, whether improvements generalize to middle-aged and older adults without injury or those participating in exercise/rehabilitation programs is unknown. Additionally, no studies have examined the impact of pain and loss of ADLs/participation in sport in conjunction with mental wellbeing. This is especially important to assess in middle-aged active males and females since they are at greater risk of age- and activity-related injury and pain compared to younger active populations or their sedentary counterparts [7]. Furthermore, these studies have evaluated dosages ranging from 10 g/d to 15 g/d with improvements in a dose response manner, but no studies have evaluated a higher dose (e.g. 20 g/d) [14–19]. Therefore, an optimal dose of CP for this population has not yet been established.

Therefore, **the purpose of this study** was to evaluate the effects of three doses (0 g/d, 10 g/d, and 20 g/d) of CP supplementation for 3, 6, and 9 months on measures of pain, function, and mental and physical health-related outcomes in middle-aged physically active males and females. We hypothesize that supplementation with 10 g/d and 20 g/d of collagen peptide will result in superior outcome measures compared to placebo, and that 20 g/d will provide greater improvements in study outcomes compared to 10 g/d.

## 2. Methods

### 2.1. Participants

**Figure 1.**
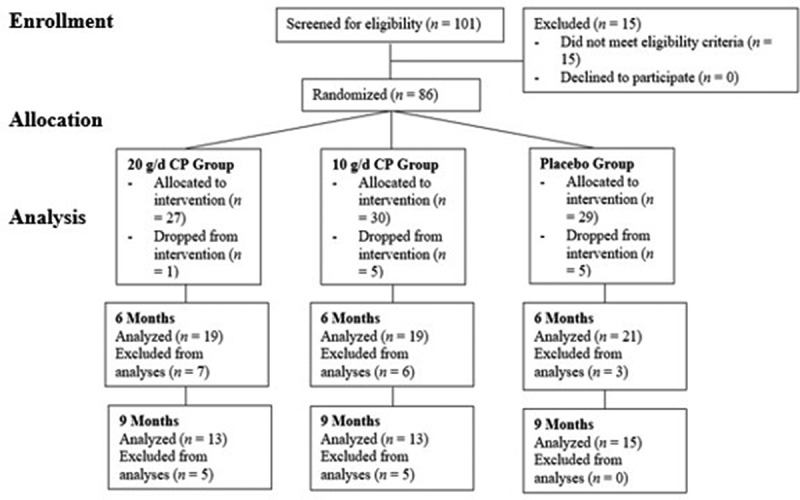
Flowchart of participant recruitment, dropouts, and randomization before and during study intervention. CP = collagen peptides, g = grams, d = day.

Eighty-six males and female life-long exercisers met study criteria and were randomized to treatment conditions described below between October 2018 and November 2019 in Tallahassee, Florida. A total of 75 participants (Male = 42; age = 54.8 ± 7.3 yrs; BMI = 26.6 ± 3.2 kg/m^2^) and (Female Male = 33; age = 54.0 ± 6.5 yrs; BMI = 23.2 ± 2.6 kg/m^2^) completed the original 6-month arm of the intervention, of which 51 remained to complete an optional additional 3-month arm of the intervention (9 months total). Of these, 59 were used for analyses of the 6-month data, and 41 for the analyses of the 9-month data. This was due to the removal of participants who were injured outside of the study protocol while enrolled in the study. After careful analysis of outliers in the present study data set, rather than removing outliers without cause, it was decided to remove participants who experienced significant injuries during study participation instead, regardless of outlier status (Figure 1). Injuries were assessed via monthly pain and injury questionnaire at each visit, and all participants who experienced injuries that caused increased pain during study participation were removed from analyses. The removal of these participants from the analyses was done prior to unblinding.

Study candidates were screened by phone (*N* = 101) to determine eligibility to participate in the study (Figure 1). Participants were eligible to participate if they were between the ages of 40–65 years, performed greater than 4 h per week of planned physical activity consistently over the past 15 years, reported having periodic but sustained/chronic joint and/or musculoskeletal pain, and currently lived in or near Tallahassee, Florida. Participants were excluded if they were already consuming CP supplements (participants could discontinue supplementation for 4 weeks to be reconsidered), were using pain medications that could not be discontinued during study enrollment, had a cortisone shot within the past 30 days, had surgery within the past 12 months, had initiated hormone replacement therapy within the last 12 months, had non-medicated hypertension or uncontrolled yet medicated hypertension, were currently smoking > 20 cigarettes per day, or had a medical diagnosis of osteoarthritis in the knee, hip, or shoulder joint, autoimmune disorder, or kidney dysfunction. Additionally, anyone with an allergy to dairy or animal products or who refused to consume these products were also excluded.

### 2.2. Experimental design

This study was a double-blind, placebo-controlled, randomized study designed to examine the effects of 3, 6, and 9 months of CP supplementation on joint function, pain, and mental/social health in older life-long physically active males and females. Participants were asked to abstain from food and caloric beverages (12 h), caffeine (12 h), alcohol (24 h), and physical activity (24 h) prior to all study visits. At baseline visit, participants reported to the Institute of Sports Sciences and Medicine (ISSM) at Florida State University (FSU), where written informed consent to participate in the study intervention was obtained and participants were randomized to one of the three treatment groups: 0 g/d (PLA), or 10 g/d or 20 g/d CP (SOLUGEL® Collagen Peptides, PB Leiner, part of Tessenderlo Group, Vilvoorde, Belgium). Participants were randomized and stratified into one of the three treatment groups by sex, using the computer-generated randomization function in Microsoft Excel (Microsoft Corporation, Redmond, WA.).

At baseline, participants completed three questionnaires evaluating pain and function, mental/social and physical health, and physical activity levels. Participants completed a three-day food record (2 weekdays and 1 weekend day) following the visit. All participants (*n* = 86) participated in five more visits separated by at least 30 days (±7 days) that were identical to the baseline visit. After the 6-month visit, participants had the option to continue the study for another three months, for a total of 9 months. Participants who agreed to the continuation (*n* = 51) signed a consent form addendum, remained in their assigned treatment group, and underwent the same evaluations as the previous six months. This study was approved by the FSU Institutional Review Board (STUDY00000044).

### 2.3. Supplementation

Participants received a 1-month supply of their respective treatments supplement packages (placebo = maltodextrin, 10 g CP, or 20 g CP) at each monthly visit along with a compliance log to track days and times of supplementation. Treatments were packaged and supplied in opaque packets by the Sponsor to maintain a double-blind design. Participants were instructed to take the CP supplement twice daily (morning and evening) to achieve their total daily dose. The empty supplement packets and compliance log were brought back to the ISSM at each subsequent visit to determine if ≥ 80% adherence to supplementation was maintained. Participant compliance of ≥ 80% was to be maintained to remain in the study, and thus was verified at each monthly visit by study staff using a two-person quality control process (no participants were disqualified for noncompliance). No adverse effects were self-reported by participants in any of the treatment groups during study participation. Therefore, the CP supplements at both doses were found to be safe and well tolerated. The CP product (SOLUGEL ®) was third party tested to ensure good manufacturing practices.

### 2.4. Surveys

At each monthly visit, participants completed The Knee Injury & Osteoarthritis Outcome Score (KOOS), to assess five separate dimensions: Pain (nine items), symptoms (Sx) (seven items), ADLs (17 items), sport and recreation function (Sport) (five items), and knee-related quality of life (QOL) (four items) [20]. All items were scored from 0 to 4, with the sum of each transformed into a 0-to-100 scale. Subscale aggregate scores of 0 represent extreme dysfunction or problems, while scores of 100 indicate normal function or no impairments [20].

The Veterans Rand 12 Item Health Survey (VR-12), a 12-item survey, was administered at each monthly visit to assess mental and physical health component scores (MCS and PCS). The MCS emphasizes mental health and social functioning, while the PCS assesses physical function and pain. VR-12 scores are standardized using a T-score metric ranging from 0-to-100, with a mean score of 50 for the general population. Increasing values indicate more favorable outcomes [21].

Physical activity was assessed at each monthly visit via the following survey questions: 1) How many minutes per week do you participate in low to moderate intensity cardiovascular training? 2) Please list the types of low to moderate intensity cardiovascular type exercises. 3) How many minutes per week do you participate in high to vigorous intensity cardiovascular training? 4) Please list the types of high to vigorous intensity cardiovascular type exercises. 5) How many minutes per week do you participate in strength training? 6) Please list other types of exercises. These questions were used to quantify minutes per week on average of the following types of exercise: 1) low to moderate intensity aerobic, 2) vigorous intensity aerobic, 3) strength training.

### 2.5. Dietary intake

Nutritional intake was assessed using a three-day food log completed for two weekdays and one weekend day after each monthly visit, using an account created in MyFitnessPal by the research staff. Diet logs were used to account for intake of total calories, carbohydrates (g/day), protein (g/day), and fat (g/day). Nutrition was not an outcome variable and was strictly assessed to ensure that no significant differences in macronutrient or kcal intake existed between groups.

### 2.6. Statistical analyses

All data were analyzed using SPSS Statistics for Windows, version 25.0 (IBM Corp., Armonk, NY), with significance level set to α = .05. Data are presented as mean values per time point, with standard error in bar charts. Data are reported as F-statistic, *p*-value, and effect size using partial eta squared (η_p_^2^). Interpretation of effect size is as follows: η_p_^2^ >0.01 (small effect), η_p_^2^ >0.06 (medium effect), and η_p_^2^ >0.14 (large effect) [22,23].

All primary outcome measures were compared across time points using a 3 (group) × 3 (time) or 4 (time) two-way mixed model ANOVA, with primary outcome measure values at each time point (baseline, 3 months, 6 months, and 9 months) used as the within factor, and group (PLA, 10 g/d, 20 g/d) as the between factor. Mauchly’s test of sphericity was used to determine sphericity for the interaction prior to analysis of variance (*p* > .05). In the event, sphericity was violated, a Greenhouse–Geisser correction for Ɛ < .75, or Huynh-Feldt correction for Ɛ > .75, was used to determine significance for the interaction term. Violations to the assumption of normality were impossible to correct with transformations, therefore analyses with violations are reported in text and were left untransformed. All significant interactions were further explored using a one-way, repeated measures ANOVA per group (PLA, 10 g/d, and 20 g/d), with primary outcome values at each time point (baseline, 3 months, 6 months, and 9 months) as the within factor, to determine if individual groups had a significant main effect for time in outcome measures. Additional pairwise comparisons were used to determine significant differences between time points within each group.

Secondary outcome measures were used to assess the moderating effects of exercise frequency and sex differences across all outcome measures. Average low-to-moderate exercise frequency (min/week) was calculated using surveys from baseline, 3 months, and 6 months, and dichotomized into low frequency (LF: <188 minutes/week) and high frequency (HF: ≥188 minutes/week) exercisers using a 50% frequency distribution. To test secondary outcome measures, all primary outcome measures were assessed via mixed-model ANOVA tests, as stated previously, then further split by, 1) LF/HF groups, and 2) sex, to determine the effects of exercise frequency and sex on primary outcome measures, respectively.

Sample size was estimated *a priori* using G*Power version 3.1.9.7 (Heinrich Heine University Dusseldorf, North Rhine-Westphalia, Germany) using data from Clark et al. (2008) to determine the effect size necessary for the outcome variable of pain [23]. Total enrollment needed and obtained based on this power analysis for the initial 6-month study end point was 86 participants enrolled and 56 completed, accounting for a 35% attrition rate [24].

## 3. Results

### 3.1. Participants

Baseline demographic data are reported for 6- and 9-month arm participants in Table 1. No significant differences were observed in baseline values between the three treatment groups in those who completed either 6- or 9-month arms of the study. Similarly, no differences in nutritional intake were observed between treatment groups for macronutrient or kilocalorie intakes (Table 2).

**Table 1.** Baseline characteristics for participants who completed the 6-month and 9-month arm of the study intervention.

Treatment Group	*N*	Age (yrs)	Height (m)	Weight (kg)	Exercise (min/wk)
**6-Month Arm**					
20 g/d	**19**	**60.0 ± 6.5**	**1.7 ± 0.1**	**74.8 ± 13.5**	**186.6 ± 75.8**
Males	11	58.8 ± 5.3	1.8 ± 0.0	83.4 ± 9.6	169.7 ± 63.9
Females	8	52.0 ± 6.1	1.6 ± 0.1	63.0 ± 8.1	209.8 ± 88.7
10 g/d	**19**	**56.1 ± 6.8**	**1.7 ± 0.1**	**70.5 ± 14.8**	**245.8 ± 172.5**
Males	7	56.1 ± 7.8	1.8 ± 0.1	86.0 ± 10.1	307.5 ± 218.2
Females	12	56.0 ± 6.5	1.6 ± 0.1	61.5 ± 7.9	209.8 ± 137.4
Placebo	**21**	**53.2 ± 7.0**	**1.7 ± 0.1**	**76.9 ± 15.2**	**237.0 ± 189.3**
Males	12	52.6 ± 7.5	1.8 ± 0.1	85.7 ± 12.2	234.7 ± 132.0
Females	9	54.0 ± 6.5	1.7 ± 0.1	65.1 ± 10.1	240.0 ± 256.1
**9-Month Arm**					
20 g/d	**13**	**54.6 ± 7.0**	**1.70 ± 0.1**	**72.4 ± 15.1**	**184.6 ± 89.8**
Males	7	58.1 ± 5.7	1.76 ± 0.0	82.9 ± 12.1	154.1 ± 71.1
Females	6	50.5 ± 6.4	1.62 ± 0.0	60.2 ± 6.2	220.2 ± 102.3
10 g/d	**13**	**55.7 ± 7.4**	**1.68 ± 0.1**	**71.7 ± 16.2**	**240.6 ± 159.5**
Males	5	54.0 ± 8.2	1.77 ± 0.1	88.8 ± 10.7	255.5 ± 175.6
Females	8	56.8 ± 7.2	1.62 ± 0.1	61.0 ± 6.8	231.3 ± 160.5
Placebo	**15**	**52.9 ± 6.7**	**1.71 ± 0.1**	**73.2 ± 12.7**	**222.9 ± 211.8**
Males	8	52.0 ± 7.2	1.74 ± 0.0	80.1 ± 9.7	219.3 ± 126.0
Females	7	54.0 ± 6.4	1.67 ± 0.1	65.4 ± 11.5	227.1 ± 293.5

All values are reported as means ±SD. Yrs = years, *m* = meters, kg = kilograms, min = minutes, wk = week, g = grams, d = day.

**Table 2.** Average macronutrient and energy intake from baseline to 6 months.

Treatment Group	CHO (g)	PRO (g)	PRO (g/kg)	FAT (g)	kcals
20 g/d	**194.3 ± 49.8**	83.9 ± 21.8	1.14 ± .24	**72.6 ± 17.6**	**1797.6 ± 396.2**
Males (*n* = 11)	203.0 ± 44.6	91.2 ± 22.5	1.12 ± .28	77.0 ± 18.8	1912.2 ± 395.1
Females (*n* = 8)	182.4 ± 57.0	73.9 ± 17.3	1.18 ± .19	66.5 ± 14.9	1640.0 ± 362.8
10 g/d	**177.9 ± 65.7**	78.2 ± 2.4	1.15 ± .32	**66.0 ± 20.8**	**1657.6 ± 481.9**
Males (*n* = 7)	224.9 ± 52.8	91.7 ± 15.2	1.11 ± .25	81.5 ± 16.0	2040.0 ± 270.0
Females (*n* = 12)	150.5 ± 57.5	7.3 ± 19.2	1.18 ± .36	57.0 ± 18.0	1434.5 ± 439.0
Placebo	**186.4 ± 81.1**	95.3 ± 33.2	1.26 ± .36	**73.4 ± 31.2**	**1861.8 ± 667.6**
Males (*n* = 12)	224.8 ± 86.7	115.2 ± 29.0	1.40 ± .39	2262.1 ± 598.0	2262.1 ± 598.0
Females (*n* = 9)	135.2 ± 30.9	68.8 ± 14.5	1.08 ± .24	1328.1 ± 248.4	1328.1 ± 248.4

All values are reported as means ±SD. CHO = carbohydrate, PRO = protein, kcals = kilocalories, g/kg = grams per kilogram of body weight, g = grams, d = day.

Although nonspecific joint pain was investigated in this study, baseline-specific joint pain afflicted 19% of our study populations. Of those suffering from joint pain, 14% reported back/lower back pain, 13% reported hip pain, 11% reported shoulder pain, 9% reported knee pain, 2% reported Achilles pain, and 2% reported rib pain.

#### 3.1.1. Survey measures: The Knee Injury & Osteoarthritis Outcome Score (KOOS)

**Figure 2.**
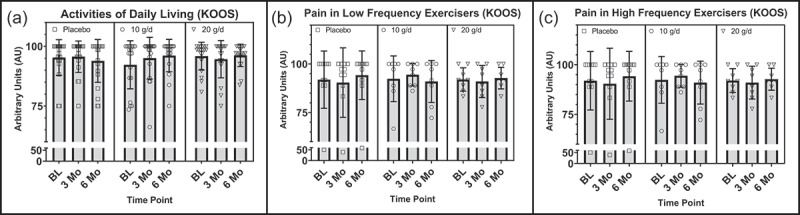
(a) Differences between treatment groups in KOOS ADLs at baseline, 3 months, and 6 months, with reported *p*-value for group by time interaction. (b) Differences between low frequency exercisers in treatment groups in KOOS Pain at baseline, 3 months, and 6 months. (c) Differences between high frequency exercisers in treatment groups in KOOS Pain at baseline, 3 months, and 6 months. AU = arbitrary units, g = grams, d = day, ADLs = activities of daily living, KOOS = Knee injury and Osteoarthritis Outcome Score, LF = low frequency, HF = high frequency.

For the survey measures of the KOOS, a significant group by time interaction was observed in ADL scores, *F*(4, 110) = 2.926, *p* = .031, η_p_^2^ = .096, from baseline to 6 months, although there was a violation of the assumption of normality and homogeneity of variance. Follow-up within group *one-way* repeated measures ANOVA tests failed to demonstrate a significant change in ADLs within groups over time, however an improvement in the 10 g/d group did approach significance with a large effect size, *F*(2, 36) = 2.839, *p* = .094, η_p_^2^ = .136 (Figure 2). No significant group by time interactions were observed in other measures of the KOOS (Pain, Sx, Sport, or QOL) when assessing 6- or 9-month study intervention end points, including ADL scores in the 9-month arm study participants.

##### 3.1.2. Secondary analyses: KOOS (sex and exercise frequency)

Although there was evidence of a group by time interaction from baseline to 9 months in males but not females in KOOS ADLs, *F*(6, 51) = 2.153, *p* = .063, η_p_^2^ = .202, this interaction did not meet conventional levels of statistical significance (*p* > .05) but did have a large effect size. No other measures of the KOOS approached or achieved statistically significant interactions when assessed by sex.

Alternatively, when assessing the moderating effects of exercise frequency, a significant group by time interaction from baseline to 6 months in pain scores was observed in HF but not LF exercisers, *F*(4, 56) = 2.756, *p* = .037, η_p_^2^ = .164, despite a violation of the assumption of normality (Figure 2). Similarly, although not significant, a group by time interaction in ADL scores from baseline to 6 months approached significance and had a large effect size in the HF but not LF exercisers, *F*(4, 56) = 2.210, *p* = .080, η_p_^2^ = .136.

Conversely, a trend toward a significant group by time interaction with a large effect size in QOL was observed in LF but not HF exercisers, from baseline to 9 months, *F*(6, 57) = 2.186, *p* = .067, η_p_^2^ = .187. The assumptions of normality were violated for both ADLs and QOL scores and are therefore at risk of a type II error. No other significant or approaching significant moderating effects of exercise between treatment groups were observed in the domains of the KOOS. Mean KOOS scores at individual time points can be found in Table 3.

**Table 3.** Average survey measure scores for the KOOS subdomains.

Parameter	Treatment Group	Baseline	3 Months	6 Months	9 Months
KOOS: ADLs	20 g/d	95.9 ± 5.8	94.6 ± 7.8	96.2 ± 4.7	95.6 ± 8.1
	Males	94.5 ± 6.9	92.6 ± 9.4	94.5 ± 5.5	91.8 ± 9.8
	Females	97.8 ± 3.4	97.7 ± 2.7	98.5 ± 1.8	100.0 ± 0.0
	LF	95.2 ± 6.7	94.9 ± 6.0	96.3 ± 5.2	95.3 ± 8.1
	HF	96.4 ± 5.4	94.4 ± 9.3	96.1 ± 4.5	95.8 ± 8.8
	10 g/d	92.3 ± 10.1	95.0 ± 8.7	96.1 ± 6.8^L^	93.9 ± 10.3
	Males	97.5 ± 4.9	99.2 ± 1.7	97.3 ± 6.0	89.1 ± 15.1
	Females	89.2 ± 11.2	92.6 ± 10.3	95.5 ± 7.4	96.9 ± 4.9*^L^
	LF	91.5 ± 10.7	95.2 ± 6.6	95.8 ± 6.3	92.9 ± 9.8
	HF	92.8 ± 10.1	94.9 ± 10.3	93.7 ± 11.2	94.7 ± 11.4
	Placebo	95.3 ± 7.5	95.7 ± 6.6	93.9 ± 9.0	98.3 ± 3.6
	Males	93.4 ± 9.3	93.6 ± 8.0	91.1 ± 10.7	97.1 ± 4.7
	Females	97.9 ± 2.8	98.4 ± 2.3	97.7 ± 3.8	99.8 ± 0.6
	LF	97.3 ± 4.1	97.5 ± 4.6	96.9 ± 7.6	98.8 ± 3.2
	HF	93.1 ± 9.8	93.7 ± 8.0	90.6 ± 9.5	97.4 ± 4.5
KOOS: Pain	20 g/d	91.5 ± 8.3	90.9 ± 9.2	92.8 ± 6.8	92.1 ± 11.5
	Males	88.6 ± 8.8	88.6 ± 10.1	94.5 ± 5.5	87.3 ± 14.0
	Females	95.5 ± 5.7	94.4 ± 7.0	96.2 ± 4.4	97.7 ± 3.7
	LF	92.0 ± 6.0	91.0 ± 8.2	92.7 ± 5.5	89.8 ± 12.5
	HF	91.2 ± 9.8	90.8 ± 10.4	92.9 ± 7.8	94.0 ± 11.2
	10 g/d	90.9 ± 12.0	91.8 ± 9.9	92.5 ± 10.8	88.7 ± 16.1
	Males	97.6 ± 5.2	95.2 ± 5.2	94.0 ± 10.2	85.0 ± 19.7
	Females	87.0 ± 13.3	89.8 ± 11.5	91.7 ± 11.5	91.0 ± 14.4
	LF	92.4 ± 11.8	94.4 ± 5.8	91.0 ± 10.8	88.4 ± 16.0
	HF	89.9 ± 10.1	89.9 ± 11.9	93.7 ± 11.2	88.9 ± 17.4^L^
	Placebo	90.9 ± 11.6	88.5 ± 14.7	89.2 ± 12.4	93.1 ± 10.7
	Males	87.3 ± 14.0	84.0 ± 17.8	85.4 ± 13.9	89.2 ± 13.5
	Females	95.7 ± 4.4	94.4 ± 5.7	87.5 ± 13.6	97.6 ± 3.4
	LF	91.9 ± 14.7	90.4 ± 17.9	94.2 ± 12.5	95.8 ± 7.9
	HF	89.7 ± 7.4	86.4 ± 10.7	83.6 ± 10.0	87.8 ± 14.4
KOOS: QOL	20 g/d	78.3 ± 17.8	80.2 **±** 18.6	81.9 ± 17.3	82.2 ± 15.5
	Males	74.4 ± 21.0	80.1 ± 21.8	75.6 ± 19.5	77.7 ± 19.7
	Females	83.6 ± 11.5	80.4 ± 13.7	90.6 ± 8.8	87.5 ± 6.8
	LF	78.9 ± 15.6	78.9 ± 24.5	82.0 ± 17.8	81.3 ± 18.5
	HF	77.8 ± 20.0	81.3 ± 13.5	84.5 ± 13.7	83.0 ± 13.8
	10 g/d	83.2 ± 15.9	76.3 ± 19.9	84.9 ± 14.9	68.8 ± 18.4
	Males	90.2 ± 11.3	83.9 ± 16.9	84.8 ± 16.9	66.3 ± 25.2
	Females	79.2 ± 17.1	71.9 ± 20.9	84.9 ± 14.5	70.3 ± 14.5
	LF	88.3 ± 11.8	77.3 ± 23.8	84.4 ± 14.9	67.7 ± 21.1*^L^
	HF	79.5 ± 17.9	75.6 ± 17.8	85.2 ± 15.6	69.6 ± 17.5
	Placebo	81.5 ± 18.2	77.4 ± 16.1	79.8 ± 18.6	81.7 ± 15.8
	Males	77.1 ± 17.3	71.4 ± 16.1	74.0 ± 20.3	74.2 ± 18.4
	Females	87.5 ± 18.5	85.4 ± 12.9	87.5 ± 10.3	90.2 ± 4.9
	LF	88.6 ± 15.5	83.0 ± 17.9	87.5 ± 18.1	86.3 ± 11.7*^L^
	HF	73.8 ± 18.4	71.3 ± 11.9	71.3 ± 15.9	72.5 ± 20.1

All values are reported as means ±SD. KOOS = The Knee Osteoarthritis & Knee Injury Outcome Score, ADLs = activities of daily living, QOL = quality of life, g = grams, d = day. *Denotes p-value < .05, for group by time interaction from mixed model ANOVA using all time points up to the time point denoted. For effect size using partial eta squared (η_p_^2^), ^S^denotes small effect size > .01, ^M^denotes medium effect size > .06, ^L^denotes large effects size > .14, for a significant or trending toward significant (*p* < .10) group by time interaction from mixed model ANOVA using all time points up to the time point denoted.

#### Survey measures: Veterans Rand 12 (VR-12)

**Figure 3.**
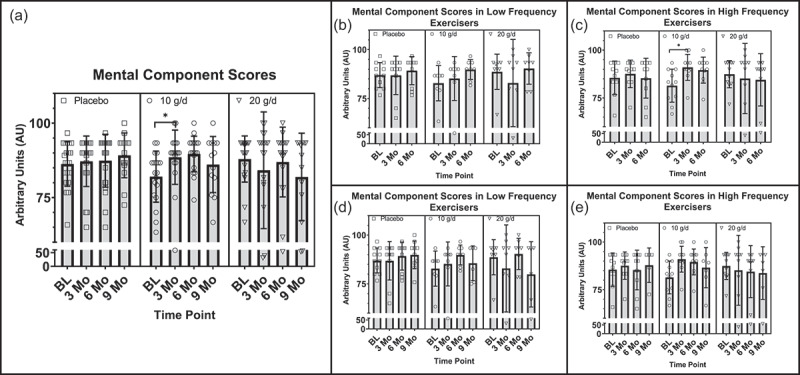
a) Differences between treatment groups in the VR-12 MCS at baseline, 3 months, 6 months, and 9 months, with reported *p*-value for group by time interaction. (b) Differences between low frequency exercisers in treatment groups in MCS at baseline, 3 months, and 6 months. (c) Differences between high frequency exercisers in treatment groups in MCS at baseline, 3 months, and 6 months, with *p*-value for group by time interaction. (d) Differences between low frequency exercisers in treatment groups in MCS at baseline, 3 months, 6 months, and 9 months. (e) Differences between high frequency exercisers in treatment groups in MCS at baseline, 3 months, 6 months, and 9 months, with *p*-value for group by time interaction. AU = arbitrary units, g = grams, d = day, MCS = mental component score, LF = low frequency, HF = high frequency.

Regarding the VR-12, a significant group by time interaction was observed in mental component score values, *F*(6, 111) = 2.685, *p* = .027, η_p_^2^ = .127, from baseline to 9 months, despite a violation of the assumption of normality. Follow-up within group *one-way* repeated measures ANOVA tests revealed a significant increase in mental component scores over time in the 10 g/d group, *F*(3, 36) = 5.371, *p* = .017, η_p_^2^ = .309, with significant increases from baseline to 3 months, *M* = −9.806, *SE* = 3.00, *p* = .04, via pairwise comparisons. No significant main effect for time on mental component scores was observed within the 20 g/d, *F*(3, 33) = .975, *p* = .381, η_p_^2^ = .081, or the PLA groups, *F*(3, 42) = 3.255, *p* = .031, η_p_^2^ = .189, when using a Bonferroni correction (α = .017), although the PLA group did approach significance with a large effect size (Figure 3). Although not significant, a trend toward a significant group by time interaction in mental component scores was observed from baseline to 6 months with a moderate effect size, *F*(4, 110) = 2.405, *p* = .064, η_p_^2^ = .080, akin to the baseline to 9-month data. These data violated the assumptions of normality and homogeneity of variances.

**Figure 4.**
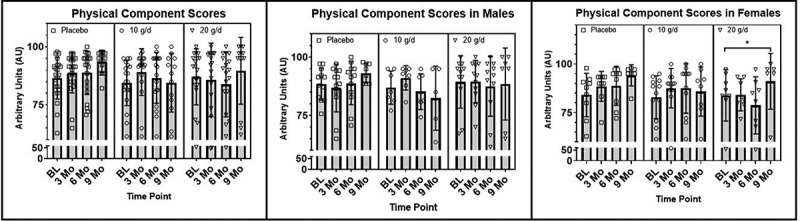
(a) Differences between treatment groups in the VR-12 PCS at baseline, 3 months, 6 months, and 9 months, with reported *p*-value for group by time interaction. (b) Differences between males in treatment groups in the VR-12 PCS at baseline, 3 months, 6 months, and 9 months. (c) Differences between females in treatment groups in the VR-12 PCS at baseline, 3 months, 6 months, and 9 months. AU = arbitrary units, g = grams, d = day, PCS = physical component score.

Additionally, a significant group by time interaction was observed in the VR-12 physical component scores, *F*(6, 111) = 2.677, *p* = .018, η_p_^2^= .126, from baseline to 9 months, despite a violation of the assumption of normality. Follow-up within group *one-way* repeated measures ANOVA tests failed to reveal significant main effects for time within the treatment groups (Figure 4).

##### 3.1.3. Secondary analyses: VR-12 (sex and exercise frequency)

While not significant, secondary analyses of sex differences did approach a significant group by time interactions with a large effect size in mental component scores in females but not males from baseline to 6 months, *F*(4, 50) = 2.473, *p* = .056, η_p_^2^= .165, and from baseline to 9 months, *F(*6, 51) = 2.313, *p* = .081, η_p_^2^= .241. It is important to note that these results are at risk of a type II error due to a violation of the assumption of normality. Similarly, a significant group by time interaction in physical component scores was observed in females but not males, from baseline to 9 months, *F*(6, 51) = 2.929, *p* = .021, η_p_^2^= .256. Follow-up one-way repeated measures ANOVA tests revealed a significant increase/improvement in the 20 g/d group (*F*(3, 12) = 5.570, *p* = .013, η_p_^2^= .582) despite a violation of normality. No significant main effects for time were observed in the 10 g/d or PLA groups Figure 4).

Furthermore, analyses of the moderating effects of exercise frequency revealed a significant group by time interaction in mental component scores in HF but not LF exercisers, from baseline to 6 months, *F*(4, 56) = 3.534, *p* = .012, η_p_^2^ = .202, despite a violation of normality. A statistically significant main effect for time in mental component scores was observed in HF exercisers in the 10 g/d group *F*(2, 20) = 8.427, *p* = .008, η_p_^2^ = .457, with a significant increase from baseline to 3 months, *M* = −9.545, SE = 2.894, *p* = .024. There were no significant main effects for time in HF exercisers in the 20 g/d or PLA groups (Figure 3). Similarly, a statistically significant interaction was observed between the interventions and time in mental component scores, *F*(6, 45) = 3.139, *p* = .012, η_p_^2^ = .295, in HF but not LF exercisers from baseline to 9 months, despite a violation of normality. However, no significant main effects for time were found in post hoc analyses within groups (Figure 3). Additionally, no significant moderating effects of exercise were found in physical component scores between treatment groups in the 6- or 9-month cohorts. Mean VR-12 scores at individual time points can be found in Table 4.

**Table 4.** Average VR-12 mental and physical component scores.

Parameter	Tx Group	Baseline	3 Months	6 Months	9 Months
VR-12: MCS	20 g/d	87.9 ± 7.6	84.2 ± 19.6	86.9 ± 11.7	81.9 ± 14.7
	Males	87.9 ± 9.9	84.2 ± 24.8	87.9 ± 12.6	78.3 ± 18.9
	Females	88.0 ± 4.0	84.2 ± 8.2	85.6 ± 11.1	86.1 ± 7.2
	LF	88.6 ± 9.0	82.9 ± 22.3	90.3 ± 8.0	79.9 ± 16.7
	HF	87.4 ± 7.1	85.2 ± 18.3	84.5 ± 13.7	83.7 ± 13.8
	10 g/d	82.0 ± 8.6	88.6 ± 9.1	89.7 ± 6.0*^L^	86.1 ± 9.4*^L^
	Males	84.2 ± 7.7	90.2 ± 7.6	88.6 ± 6.6*^L^	87.2 ± 5.8
	Females	80.8 ± 9.1	87.6 ± 10.1	90.3 ± 5.8^L^	85.4 ± 11.5
	LF	82.8 ± 9.0	85.2 ± 11.2	89.8 ± 5.1	85.6 ± 8.9^L^
	HF	81.4 ± 8.6	91.0 ± 6.8	89.5 ± 6.8*^L^	86.6 ± 10.5*^L^
	Placebo	86.3 ± 7.5	87.2 ± 8.5	87.4 ± 8.9	89.2 ± 7.5*^L^
	Males	84.9 ± 7.9	86.8 ± 10.3	88.1 ± 8.1	87.7 ± 6.9*^L^
	Females	88.1 ± 6.9	88.5 ± 7.7	86.5 ± 10.3	90.8 ± 8.3
	LF	87.0 ± 6.4	86.8 ± 9.4	89.2 ± 7.2	89.8 ± 7.1
	HF	85.5 ± 8.8	87.6 ± 7.2	85.3 ± 10.4	87.8 ± 14.4
VR-12: PCS	20 g/d	87.1 ± 12.1	85.8 ± 15.8	83.8 ± 13.9	89.7 ± 14.4
	Males	89.4 ± 11.2	89.5 ± 9.0	87.5 ± 12.8	88.5 ± 15.4
	Females	83.9 ± 13.2	79.8 ± 22.5	78.8 ± 14.7	91.1 ± 14.3*^L^
	LF	90.4 ± 6.4	88.8 ± 16.0	87.1 ± 9.9	92.9 ± 13.8
	HF	84.6 ± 14.8	83.4 ± 16.1	81.4 ± 16.3	86.9 ± 15.3
	10 g/d	84.4 ± 9.7	89.1 ± 10.0	86.6 ± 10.9	84.6 ± 12.6
	Males	86.9 ± 7.1	91.1 ± 5.2	85.2 ± 7.6	82.4 ± 13.8
	Females	82.9 ± 11.0	88.0 ± 12.1	87.4 ± 12.7	85.9 ± 12.7
	LF	83.0 ± 11.9	85.2 ± 14.0	87.3 ± 11.9	81.1 ± 16.0
	HF	85.4 ± 8.3	92.0 ± 4.5	86.1 ± 10.7	87.6 ± 9.1
	Placebo	86.5 ± 9.0	88.7 ± 8.6	88.8 ± 9.3	93.6 ± 4.7^L^
	Males	88.4 ± 6.9	86.8 ± 10.3	88.8 ± 9.2	93.1 ± 4.2
	Females	84.0 ± 11.1	91.3 ± 5.0	88.8 ± 10.0	94.2 ± 5.4
	LF	87.6 ± 8.9	89.3 ± 9.4	90.1 ± 9.9	93.8 ± 5.0
	HF	85.4 ± 9.4	88.1 ± 7.9	87.4 ± 8.9	93.3 ± 4.4

All values are reported as means ±SD. Tx = treatment, VR-12 = The Veterans Rand 12, MCS = mental component score, PCS = physical component score, g = grams, d = day. *Denotes p-value < .05, for group by time interaction from mixed model ANOVA using all time points up to the time point denoted. For effect size using partial eta squared (η_p_^2^), ^S^denotes small effect size > .01, ^M^denotes medium effect size > .06, ^L^denotes large effects size > .14, for a significant or trending toward significant (*p* < .10) group by time interaction from mixed model ANOVA using all time points up to the time point denoted.

## 4. Discussion

This was the first study to investigate the effects of supplementing with two doses of CP compared to PLA on pain, function, and mental/social as well as physical health-related QOL over 3, 6, and 9 months in middle-aged physically active males and females. The main findings from this study are that significant improvements in ADLs and pain, measured via the KOOS, were observed with 10 g/d of CP supplementation over 6 months, although pain only improved in HF exercisers. Similarly, improvements in the VR-12 mental component scores were observed after supplementing with 10 g/d over 6 and 9 months. Further secondary analyses also demonstrated improvements with 10 g/d of CP over 9 months in females, but not males, and in HF but not in LF exercisers. Lastly, physical components scores of the VR-12 significantly improved with 20 g/d over the course of 9 months in females, but not in males, with no differences in this measure observed between HF and LF exercisers.

Our findings of improvements in ADLs and pain are in accordance with prior literature reporting increased return to ADLs, improvements in pain, as well as accelerated return to play after sports related injuries [14–19]. For example, Dressler et al. (2018) reported significantly decreased pain and increased function in college age athletes with chronic ankle strains after supplementing with 5 g/d of CP with an at home rehabilitative program over 6 months when compared to PLA [25]. In a similar population, significant improvements in activity-related pain were observed in college age athletes after 6 months of supplementation with 10 g/d of CP compared to PLA [18]. Likewise, in an older population, 1.2 g/d of CP supplementation over 6 months reduced pain in the most painful joint in adults ages 50 years or older, when compared to PLA [9]. Also, older adults suffering from Achilles tendon strains reported significantly decreased pain and faster return to play/ADLs after supplementing with 5 g/d CP with an at home rehabilitative program over 3 months, when compared to a PLA [26,27]. To summarize, oral supplementation of CP in conjunction with rehabilitative exercise programs have been reported to accelerate recovery from Achilles and patellar tendinopathies, and ankle instability, compared to controls [16,25,27].

Akin to studies by Praet et al. (2019) and Bruyère et al. (2012), the current study population reported improvements in the KOOS measure for pain in those exercising at a low to moderate intensity greater than 188 min per week. This indicates that frequent physical activity may moderate the effects of the CP supplementation on pain. The moderating effects of exercise on mitigating pain with CP supplementation may be attributed to increased blood flow and delivery of amino acids and bioactive peptides to the connective tissue during exercise, which is otherwise poorly vascularized [28–30]. The presence of these building blocks can be especially useful in exercise due to upregulation of collagen turnover stimulated by force production during exercise as well [29]. Interestingly, the 50% frequency distribution cutoff in our study for high and low exercise frequency groups (HF and LF) was similar to the minimum physical activity recommendations outlined in the Physical Activity Guidelines for Americans of 150–300 min a week of moderate-intensity aerobic exercise [31]. Therefore, CP supplementation may especially benefit those with chronic pain who are meeting the minimum recommendations for physical activity. The present study demonstrated smaller, but not significant, improvements in pain scores from baseline to 6 months in the 10 g/d group compared to the 20 g/d group, suggesting a possible point of diminishing returns near the 20 g/d dose, while 10 g/d may be more optimal for improving pain and ADLs. Interestingly, as pain decreased in the HF exercise treatment groups, a concomitant increase in pain was observed in the PLA group which coincides with literature reporting a strong association between pain and higher frequency of exercise [7]. Therefore, the magnitude of improvement in chronic pain in both the treatment groups may have been blunted by offsetting the pain associated with exercise. Our findings indicate that CP supplementation may have a protective effect as well as a pain mitigating effect in those exercising >188 min/week.

Although effective at 6 months, the benefits of CP supplementation on ADLs and pain measured at 6 months were not present when measured at 9 months. This could be due to fewer participants remaining in the 9-month arm of the study, or to the exclusion of participants in analyses due to injuries. Nevertheless, although not significant, a trend toward a significant group by time interaction with a large effect size in ADLs was observed in HF exercisers at 9 months (*p* = .080, η_p_^2^ = .136). This trend showed a non-significant improvement in ADL scores in the 10 g/d group, which would indicate improved function in participants meeting the exercise guidelines for Americans (>188 min/week). Additionally, although ADL scores remained unchanged from baseline to 9 months in the 20 g/d group, there was a trend toward a decline in the PLA group. This could mean that supplementation with 20 g/d could have a protective effect in the maintenance of ADLs, while 10 g/d may be more optimal for inducing improvements, although this is purely speculative. The improvements at a lower dose of supplementation could be attributed to higher levels of glycine initiating a positive feedback loop for shuttling glycine into the urea cycle, thereby decreasing transcription signaling or lessening the building blocks available for new collagen synthesis [32], although this is purely speculative.

Contrary to our other study findings, a treatment by time interaction in QOL scores approached significance with a large effect size in LF but not HF exercisers, from baseline to 9 months (*p* = .067, η_p_^2^ = .187). These decreases in QOL in the LF exercisers may be independent of the treatment, and more likely a reflection of how LF of physical activity may diminish QOL [33]. However, exploratory *one-way* repeated measures analyses showed a significant decline from baseline to 9 months in QOL in the 10 g/d and PLA groups, while the 20 g/d group did not significantly change (Table 3). This could suggest a protective affect against these decrements in LF exercisers when supplementing with 20 g/d for 9 months.

To our knowledge, this is the first study to examine the effects of CP supplementation on the VR-12 and to also find significant improvements in mood with supplementation. Moreover, limited studies have evaluated the impact of CP supplementation on mood or mental health, and those that have, did not yield effects. For example, Clifford et al. (2019) examined the effects of 12 weeks of CP supplementation on the Brief Assessment of Mood Adapted (BAM+) in young recreationally active males and found no significant differences between the CP treatment group and control [34]. Alternatively, Nogimura et al. (2020) found that CP supplementation in mice resulted in suppressed depressive symptoms in response to forced swimming when compared to PLA group [35]. Similarly, Mizushige et al. (2019) reported improvements in depression in response to CP supplementation in non-exercising and non-stressed mice (sans forced swim) when compared to PLA [36]. Furthermore, in both, the Nogimura et al. (2020) and Mizushige et al. (2019) studies, greater concentrations of Pro-Hyp compared to Hyp-Gly were found in the blood after CP supplementation compared to controls, which interestingly was found in the cerebral spinal fluid (CSF) and the brain, while the other bioactive peptide tested, Hyp-Gly, was not [35,36]. These findings indicate that certain bioactive peptides can pass through the blood brain barrier, while others cannot, and may therefore exert effects on specific tissue types (i.e. regions of the brain) [35,36]. Furthermore, decreased depressive symptoms were coupled with upregulated gene expression of neurotropic factors in the hippocampus and with increased dopamine secretion in the prefrontal cortex [35,36]. Therefore, the improved mood associated with CP supplementation may be due to increased secretion of neurotropic factors and neurotransmitters [35,36].

Correspondingly, cognitive function and brain structure in humans have also shown to benefit from CP supplementation. In a clinical pilot study, Koizumi et al., (2019) found that CP supplementation of 5 g/d for 4 weeks improved cognitive function and memory, as well as brain structure, measured by fMRI, in healthy participants ages 49 to 63 years [37]. This study also evaluated mental component scores (MCS) and physical component scores (PCS) via the SF-36, which is similar to the VR-12, but found no significant change from baseline to 4 weeks. Although not significant in their study, mental component scores did trend toward a significant improvement with 5 g/d for 4 weeks [37], akin to our 9 months study findings with 10 g/d. Importantly, this study lacked a control group for comparison, so their findings were based on differences in measures from baseline to 4 weeks across the entire sample population [37].

As previously stated, pain-related decreases in function can lead to decrements in mood and mental health, resulting in a cycle of restriction [5,8,38]. Hence, it is possible the improvement in mental component scores observed in the 10 g/d group at 9 months in our study were a result of improved ADLs observed at 6 months in the 10 g/d group. Additionally, pain decreased in the HF exercisers in the 10 g/d group, which also corresponds with the improved mental component scores we observed at 9 months.

The second component of the VR-12, the physical component score, is a measure of physical QOL. A significant group by time interaction in physical component scores was observed at 9 months, and although no significant main effects for time were observed between groups in post hoc analyses, females in the 20 g/d group had significant improvements in physical component scores, whereas those in the 10 g/d and PLA groups did not. These findings suggest that the interaction observed in the total sample at 9 months was driven by females and not males, and that 20 g/d over 9 months had a significantly beneficial effect on the VR-12 physical component scores in females.

### 5.1. Limitations

It is important to note these data were not normally distributed and violated the assumptions of homogeneity of variances and are therefore at risk of type II errors. This may mask potentially significant results, and therefore the *p*-values for these data should be coupled with their partial eta squared (η_p_^2^) values when interpreting their results. Additionally, as the current study was conducted in free living active adults, rather than during a structured exercise program, variability in exercise associated outcomes was high. Alternatively, this may be considered a strength, as a freely exercising study population translates more readily to the general population of physically active middle-aged and older adults. Furthermore, significant findings are more difficult to detect in such a free-living population, resulting in increased confidence in the efficacy of our study outcome measures that were significantly improved with CP supplementation. Moreover, because the study was extended to 9 months after participant recruitment, not all participants elected to remain in the study, which reduced our sample size in the 9-month arm. This resulted in loss of power, especially when assessing the moderating effects of exercise and sex differences. Finally, this study was powered on the effects of CP supplementation on pain over 6 months; therefore, power for the other assessments tested may not be adequate, and the risk of type II error in these outcome measures may be increased.

## 6. Conclusions

Together, these findings suggest that 10 g/d of CP supplementation may be superior to a larger dose of 20 g/d, but both doses are better than nothing at all in improving physical function/ADLs, pain, mental component scores, and physical component scores in those supplementing for at least 6 months. Additionally, females may see improvements at higher doses than males, and higher frequency exercisers may experience enhanced benefits compared to their more sedentary counterparts. Finally, declines in quality of life (QOL) were observed over 6 months of supplementation in more sedentary participants in the 10 g/d and placebo groups, but declines were not as large in participants taking 20 g/d of CP, suggesting a larger dose may be needed to protect against declines in QOL over time. Further, as evidenced by our data, the effects of collagen supplementation may be outcome, time, dose, and population dependent.
